# Risk factors and their diagnostic values for ocular metastases in invasive ductal carcinoma

**DOI:** 10.1002/cam4.3656

**Published:** 2020-12-18

**Authors:** Rong‐Bin Liang, Kang Yu, Jie‐Li Wu, Jia‐Xiang Liu, Qi Lin, Biao Li, Yu‐Qing Zhang, Qian‐Min Ge, Qiu‐Yu Li, Hui‐Ye Shu, Yi Shao

**Affiliations:** ^1^ Department of Ophthalmology The First Affiliated Hospital of Nanchang University Nanchang Jiangxi China; ^2^ Department of Ophthalmology Xiang'an Hospital of Xiamen University Fujian Provincial Key Laboratory of Ophthalmology and Visual Science Eye Institute of Xiamen University Xiamen University School of Medicine Xiamen Fujian Province China

**Keywords:** diagnostic value, HIF‐1, invasive ductal cancer, ocular metastasis, risk factors

## Abstract

Invasive ductal carcinoma (IDC) is a major type of breast cancer. Ocular metastasis (OM) in IDC is rarely seen, but patients with OM often have a poor prognosis. Furthermore, OM is difficult to detect in the early stages by common imaging examinations. In the present study, we tried to figure out the risk factors of OM in IDC and evaluate their diagnostic values for early detection. There were 1192 IDC patients who were divided into two groups according to ocular metastasis involved in this study. Clinical parameters of those patients were used to detect differences. The binary logistic regression test was then used to determine the risk factors of OM in IDC. Furthermore, ROC curves of both single and combined risk factors were established to examine their diagnostic values. The incidence of axillary lymph node metastases was significantly higher in the OM group (*p* = 0.002). Higher carbohydrate antigen 153 (CA153), lower apolipoprotein A1 (ApoA1), and hemoglobin (Hb) were risk factors for OM in IDC (*p* < 0.001, *p* < 0.001, *p* = 0.038, respectively). In the single risk factor ROC analysis, cutoff values of CA153, ApoA1, and Hb were 43.3 u/mL (CI: 0.966–0.984, *p* < 0.001), 1.11 g/L (CI: 0.923–0.951, *p* < 0.001), and 112 g/L (CI: 0.815–0.857, *p* < 0.001), respectively. Among the ROC curves of combined risk factors, CA153+ApoA1+Hb had the best accuracy, with the sensitivity and specificity of 89.47% and 99.32%, respectively (CI: 0.964–0.983, *p* < 0.001). CA153, ApoA1, and Hb are risk factors for OM in IDC. In clinical practice, the three parameters could be used as predictive factors for the early detection of OM.

## Graphical abstract

The study provided a new method in the preventive diagnosis of distant metastasis in breast cancer. Risk factors CA153, ApoA1, and Hb could be used in clinical practice to predict OM in patients with IDC.

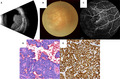

## 1 INTRODUCTION

Breast cancer has become common cancer in female and invasive ductal carcinoma (IDC), a major type of it, accounts for approximately 72% of all breast cancer.^1^, ^2^ IDC originates from ductal epithelium and is formed when tumor cells penetrate the basement membrane of normal mammary ducts or acini and invade the breast stroma. Because of its invasive characteristic, IDC is prone to metastasis through either the lymphatic pathway or blood circulation pathway. Moreover, breast cancer is the main cause of ocular metastasis (OM).^3^ Though not common, OM can present a series of severe symptoms such as foreign body sensation, ocular pain, visual field defects, and even blindness. In clinical practice, however, physicians often focus on the primary tumor, failing to notice the potential signs of OM at an early stage. When OM progresses to an advanced stage, ophthalmectomy is often needed as chemotherapy drugs are not easy to reach the eyes, which always means disasters for patients.

Although there are abundantly available treatment options such as surgery, chemotherapy, radiotherapy, and targeted therapy, OM patients always have a poor outcome because of the particularity of the metastatic site.

The detection methods which are commonly used include ocular ultrasound, fundus photography, fundus angiography, magnetic resonance imaging (MRI), and computed tomography (CT). However, when OM is detected, the disease has always developed into an advanced stage. Hence, it is imperative to diagnose early OM in patients with IDC to take effective measures in time.

Hematological indices of patients sometimes can reflect the progress of their diseases earlier than imaging examination. The method of analyzing clinical features and hematological indices of patients has already been used to evaluate the possibility of distant metastases.^4^, ^5^, ^6^, ^7^ As the method is non‐invasive and repeatable, it is useful in clinical practice to identify distant metastases in an early stage.

In this retrospective study, we tried to find out risk factors and their diagnostic values for OM in IDC through analyzing the clinical features and hematological indices of IDC patients.

## 2 MATERIALS AND METHODS

### 2.1 Data collection

**Figure 1:**
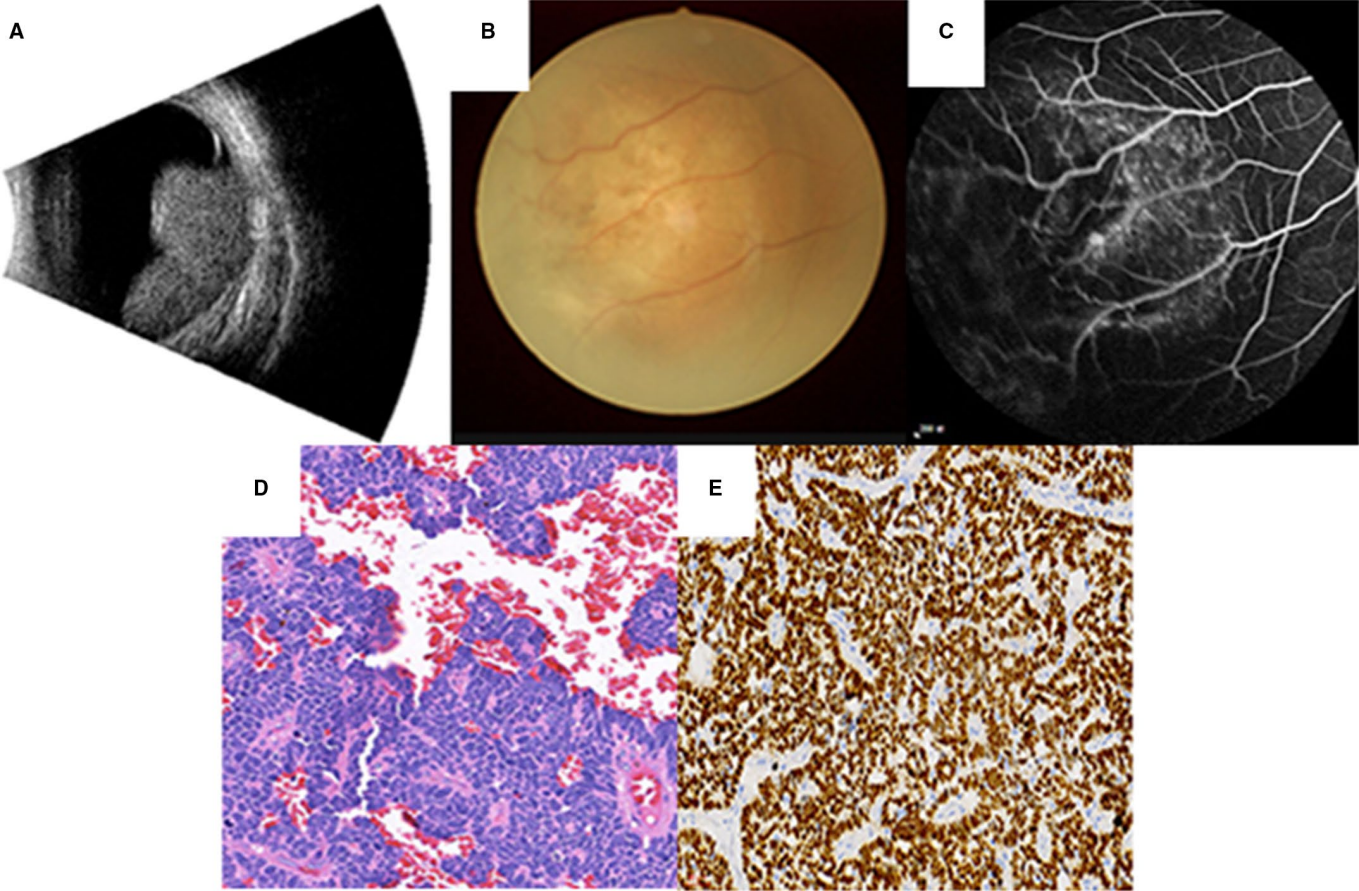
Imaging and pathological data of OM in IDC patients. (A) Ultrasound examination of ocular (right eye). Hypoechoic mass with an irregular shape could be seen in the posterior pole of the right eye. Echo attenuated behind the mass and infiltration could be seen around the mass. (B) Fundus photography (right eye). Under the retina, yellow nodular flat bulges with unclear boundaries could be seen. (C) Fundus angiography (right eye). Fluorescence leakage and low fluorescence in the lesion could be seen. Blood vessels were visualized in the field of vision. (D) Hematoxylin‐eosin staining of the right ocular tumor, X200. Infiltration of cancer cells arranged in cords and nests with hyperchromatic nuclei could be seen in the tissue. Cellular atypia was obvious.

A total of 1192 patients with IDC were involved in this retrospective study. The samples were collected from the year 2008 to 2017 in the medical records systems. The inclusion criteria were as follows: (i) Patients were diagnosed with primary breast cancer at The First Affiliated Hospital of Nanchang University. (ii) Ophthalmic examinations in OM patients indicated intraocular space‐occupying lesions. (iii) The pathological diagnosis of IDC was confirmed by the Pathology Department of The First Affiliated Hospital of Nanchang University. In addition, patients with metastatic breast cancer or primary ocular malignant tumor were excluded from the study. Imaging data and pathological results of patients are shown in Figure 1.

The clinical parameters of the patients were compiled from their medical records including age, menopausal status, axillary lymph node metastases (ALNM), triglycerides (TG),total cholesterol (TC), high‐density lipoprotein (HDL), low‐density lipoprotein (LDL), apolipoprotein A1 (ApoA1), apolipoprotein B (ApoB), lipoprotein A (LipA), alkaline phosphatase (ALP), calcium (Ca) carcinoembryonic antigen (CEA), carbohydrate antigen 125 (CA125), carbohydrate antigen 153 (CA153), and carbohydrate antigen 199 (CA199). Continuous data were presented as mean ±standard deviation. This study meets the requirements of the declaration of Helsinki and was approved by the Ethics Committee of The First Affiliated Hospital of Nanchang University.

### 2.2 Study design

All patients were divided into two groups: the OM group and the non‐ocular metastasis (NOM) group. The diagnoses of IDC and OM were achieved by biopsy. The clinical features of the patients between the OM and NOM groups were compared to find out the differences. Significant features were evaluated through binary logistic regression to figure out the risk factors of OM in IDC patients. The receiver operating characteristic (ROC) curves were then established to assess the diagnostic accuracy of the risk factors in clinical practice.

### 2.3 Statistical analysis

First, the chi‐squared test was used to detect differences in age, menopausal status, and ALNM. The normality test was then performed to determine the normal distribution of the data. As the data did not obey the normal distribution, clinical parameters between the two groups were compared using the rank‐sum test. Binary logistic regression was used to determine the risk factors for OM in patients with IDC. ROC curves of a single risk factor were established to evaluate the predictive accuracy. ROC curves of combined risk factors were also established to determine the best method of diagnosing OM in IDC patients. The ROC curves of combined factors were established in the following steps: (i) The binary logistic regression analysis was firstly made using the combined factors as covariates and using the status (whether developing OM) as dependent variables. (ii) Then obtain the prediction probabilities of each sample and put them in the ROC analysis as the values of the specific test (Using the combined factors to detect OM was defined as a test). The SPSS 17.0 (SPSS, IBM Corp, USA) software was used to process the data.

## 3 RESULTS

### 3.1 Demography and clinical features

There were a total of 1192 patients with IDC (19 with OM, 1173 without OM). In the OM group, the mean age was 44.79 ± 7.91 years, whereas the age in the NOM group was 48.06 ± 10.32 years. In the OM group, 15 patients were premenopausal and 4 were postmenopausal, while in the NOM group, 732 patients were premenopausal and 441 were postmenopausal (*p *= 0.139). There were 728 patients with ALNM (16 OMs vs 712 NOMs) and 464 patients without ALNM (3 OMs vs 461 NOMs) (*p *= 0.001) (Table 1). There were no differences in age and menopausal status. However, the incidence of OM rates showed an increasing trend with increasing number of lymph node metastasis.

**Table 1:** Clinical features of patients with invasive ductal carcinoma.

	OM	NOM	χ^2^ value	*p* value
Age (years)	44.79 ± 7.91	48.06 ± 10.32	1.134	0.649
<25	0	5		
25–65	19	1108		
>65	0	60		
Menopausal status (n)				
Premenopausal	15	732	2.187	0.139
Postmenopausal	4	441		
Axillary lymph node metastases (n)				
0	3	461	12.204	0.002
1–4	4	417		
>4	12	295		
ER				
Positive	12	741	0.621	0.733
Negative	6	403		
Unknown	1	29		
PR				
Positive	10	650	0.618	0.734
Negative	9	494		
Unknown	0	29		
HER2				
Positive	8	553	0.844	0.656
Negative	11	588		
Unknown	0	32		
T classification				
T1	1	107	17.957	0.001
T2	15	748		
T3	1	72		
T4	2	13		
Unknown	0	233		
Clinical stages				
I	0	73	20.275	0.001
II	7	637		
III	10	219		
IV	2	35		
Unknown	0	209		

Notes

*p* < 0.05 represented statistical significance. T1: tumor size ≤20 mm; T2: 20 mm < tumor size ≤50 mm; T3: tumor size >50 mm; T4: tumor invaded chest wall or skin. T‐classification and clinical stages were in line with the 2018 edition of the 8th AJCC TNM breast cancer staging.

Abbreviations: ER, estrogen receptor; HER2, human epidermal growth factor receptor‐2, T classification, tumor size classification; NOM, non‐ocular metastases; OM, ocular metastases; PR, progesterone receptor.

### 3.2 Risk factors of OM in patients with IDC

Rank sum test was used and significant differences were found in the levels of CEA, CA125, CA153, Hb, ALP, TC, LDL, and ApoA1 (Table 2). The result of binary logistic regression showed that IDC patients with higher level of CA153, lower level of ApoA1 and Hb were more easily to develop OM. (Table 3).

**Table 2:** Differences of clinical parameters between two groups.

Clinical features	OM group	NOM group	Z value	*p* value
CEA (ng/mL)	20.17 ± 46.97	4.70 ± 27.91	−3.539	<0.001
CA125 (u/mL)	78.84 ± 136.58	18.50 ± 40.52	−4.786	<0.001
CA153 (u/mL)	143.26 ± 131.55	17.20 ± 23.85	−7.135	<0.001
CA199 (u/mL)	25.06 ± 48.18	17.12 ± 19.53	−1.272	0.203
Hb (g/L)	99.89 ± 16.48	120.96 ± 13.43	−5.044	<0.001
ALP (u/L)	110.95 ± 75.01	65.75 ± 30.26	−3.563	<0.001
Ca (mmol/L)	2.32 ± 0.43	2.44 ± 0.58	−0.259	0.796
TC (mmol/L)	4.24 ± 0.99	5.40 ± 1.93	−2.993	0.003
TG (mmol/L)	1.55 ± 0.51	2.23 ± 1.91	−0.796	0.426
HDL (mmol/L)	2.93 ± 3.22	2.16 ± 1.72	−0.236	0.813
LDL (mmol/L)	1.84 ± 0.68	3.46 ± 1.81	−5.279	<0.001
ApoA1 (g/L)	0.90 ± 0.32	1.75 ± 0.78	−6.564	<0.001
ApoB (g/L)	1.10 ± 0.36	1.50 ± 1.26	−0.231	0.817
LipA (mg/L)	140.05 ± 30.44	185.11 ± 203.38	−0.739	0.46

Notes

Mann‐Whitney test was used. *p* < 0.05 represented statistical significance.

Abbreviations: ALP, alkaline phosphatase; ApoA1, apolipoprotein A1; ApoB, apolipoprotein B; Ca, Calcium; CA125, carbohydrate antigen 125; CA153, carbohydrate antigen 153; CA199, carbohydrate antigen 199; CEA, carcinoembryonic antigen; Hb, hemoglobin; HDL, high‐density lipoprotein; LDL, low‐density lipoprotein; LipA, lipoprotein a; NOM, non‐ocular metastases; OM, ocular metastases; TC, total cholesterol; TG, triglyceride.

**Table 3:** Binary logistic regression results.

Factors	B	Exp(B)	95% CI	*p* value
CA153 (u/mL)	0.022	1.022	1.010–1.034	<0.001
ApoA1 (g/L)	−8.169	0.000	0.000–0.011	<0.001
Hb (g/L)	−0.046	0.955	0.915–0.998	0.038

Notes

The forward‐LR method was used in the binary logistic regression analysis. *p *< 0.05 represented statistical significance.

Abbreviations: ApoA1, apolipoprotein A1; B, coefficient of regression, CI, confidence interval; CA153, carbohydrate antigen 153; Hb, hemoglobin.

### 3.3 ROC curve analysis for a single risk factor

**Figure 2:**
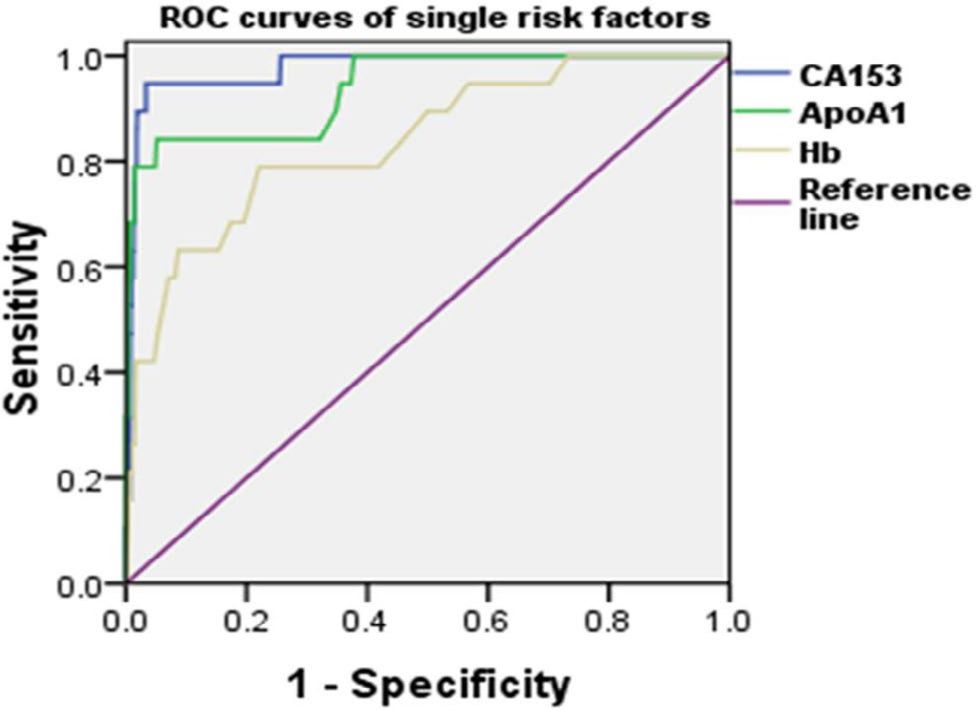
ROC curves of single risk factors for OM in IDC. ROC curves of CA153, ApoA1, and Hb are shown in the figure. Abbreviations: ROC, receiver operating characteristic; OM, ocular metastases; IDC, invasive ductal carcinoma; CA153, carbohydrate antigen 153; ApoA1, apolipoprotein A1; Hb, hemoglobin.

ROC curves were constructed for a single risk factor to evaluate their predictive values in clinical practice (Figure 2). The AUC of CA153 was 0.977 (95% CI: 0.966–0.984) and the cutoff value of CA153 was 43.3 u/mL, which means that IDC patients with CA153 levels higher than 43.3 u/mL are more likely to have OM. The sensitivity and specificity of CA153 were 94.74% and 96.68%, respectively. The AUC of ApoA1 was 0.938 (95% CI: 0.923–0.951) and the sensitivity and specificity were 84.21% and 94.88%, respectively. IDC Patients with their ApoA1 levels lower than 1.11 g/L are more likely to have OM. For Hb, the AUC was 0.837 (95% CI: 0.815–0.857) and the cutoff value was 112 g/L, with a sensitivity and specificity of 78.95% and 77.92%, respectively. To sum up, CA153 level higher than 43.3u/mL, ApoA1 level lower than 1.11g/L and Hb level less than 112g/L were threeingle risk factors for IDC patients to develop OM. Among the three single risk factors, CA153 level higher than 43.3u/mL had the best predictive accuracy (Table 4).

**Table 4:** ROC analysis of risk factors for predicting OM in IDC.

	Cutoff value	Sensitivity (%)	Specificity (%)	AUC	CI	*p*
CA153 (u/mL)	43.3	94.74	96.68	0.977	0.966–0.984	<0.001
ApoA1 (g/L)	1.11	84.21	94.88	0.938	0.923–0.951	<0.001
Hb (g/L)	112	78.95	77.92	0.837	0.815–0.857	<0.001
CA153+Hb	‐	89.47	93.78	0.97	0.959–0.979	<0.001
ApoA1+Hb	‐	84.21	98.55	0.954	0.940–0.965	<0.001
CA153+ApoA1	‐	89.47	97.78	0.968	0.957–0.977	<0.001
CA153+ApoA1+Hb	‐	89.47	99.32	0.975	0.964–0.983	<0.001

Notes

*p* < 0.05 represented statistical significance.

Abbreviations: ApoA1, apolipoprotein A1; AUC, area under the curve, CI, confidence interval; CA153, carbohydrate antigen 153; Hb, hemoglobin; ROC, receiver operating characteristic.

### 3.4 ROC curve analysis for combined risk factors

**Figure 3:**
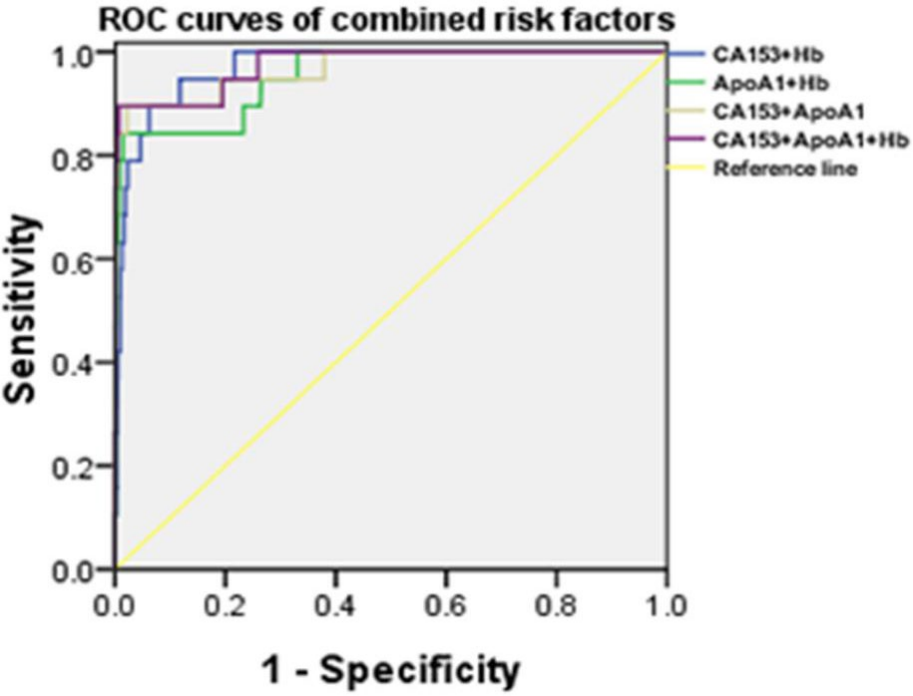
ROC curves of combined risk factors for OM in IDC. ROC curves of CA153+Hb, ApoA1+Hb, CA153+ApoA1, and CA153+ApoA1+Hb are shown in the figure. Abbreviations: ROC, receiver operating characteristic; OM, ocular metastases; IDC, invasive ductal carcinoma; CA153, carbohydrate antigen 153; ApoA1, apolipoprotein A1; Hb, hemoglobin.

To look for the best diagnostic method, ROC curves of the combined risk factors were established (Figure 3). The AUC of CA153+Hb was 0.97 (95% CI: 0.959–0.979) and the sensitivity and specificity were 89.47% and 93.78%, respectively. For ApoA1+Hb, the AUC was 0.954 (95% CI: 0.940–0.965) and the ROC curve showed a higher specificity of 98.55%, but lower sensitivity of 84.21%. For CA153+ApoA1, the AUC was 0.968(95% CI: 0.957–0.977) and the sensitivity and specificity were 89.47% and 97.78%, respectively, which were the highest among all the combined two factors. The combination of all three risk factors had the best predictive value, with the sensitivity of 89.47% and the specificity of 97.78%, and the AUC of 0.975 (95% CI: 0.964–0.983) (Table 4).

## 4 DISCUSSION

Breast cancer has become the most common cancer in women. It is a heterogeneous disease with different clinical characteristics in different subtypes.^8^

IDC is the major type of invasive nonspecific cancer, where cancer cells can easily enter the vein via the lymphatic route, or directly enter the blood circulation leading to distant metastasis.

OM is caused by hematogenous metastasis of tumor. Choroid is the most common site in an ocular where OM occurs, for the blood supply of choroid is supported by a plurality of large ciliary posterior short arteries in the posterior part of the eyeball, and there is extensive anastomotic communication between the choroidal vessels. As the blood flow of choroid is rich, it is prone to develop metastasis.

Although CT and MRI are common techniques to detect distant metastasis, they have the disadvantages of high expenditure and high radiation doses.

Recently, serum parameters and clinical features of patients have been widely applied to evaluate the possibility of metastases. Risk factors obtained by the statistical analysis were useful to predict distant metastases in clinical practice. Studies of risk factors for various metastases are summarized in Table 5.^5^, ^6^, ^9^, ^10^, ^11^, ^12^

**Table 5:** Summary literatures.

Author	Year	Primary cancer	Metastasis	Risk factors
Cao et al^5^	2012	Breast cancer	Liver	LDH+GGT+CA153
Zhou et al^11^	2017	Lung cancer	Bone	CA125+ALP
Chen et al^6^	2017	Breast cancer	Bone	ALMN+CA153+ALP+Hb
Chen et al^9^	2017	Renal cell cancer	Bone	ALP+Ca+Hb
Huang et al^10^	2017	Bladder cancer	Bone	Ca+ALP+Hb
Zhu et al^12^	2019	Colorectal cancer	Eye	HDL

Abbreviations: ALMN, axillary lymph node metastases; ALP, alkaline phosphatase; Ca, calcium; CA153, carbohydrate antigen 153, CA125, carbohydrate antigen 125; GGT, γ‐glutamyltransferase; Hb, hemoglobin; HDL, high‐density lipoprotein; LDH, lactate dehydrogenase.

In the present retrospective study, we explored the risk factors for OM in 1192 patients with IDC and analyzed their diagnostic values. As patients in the OM group were more likely to have other metastases and their diseases were also in advanced stages, they often had poor prognosis. Axillary lymph node metastasis is more common in patients with poor prognosis, which might explain the fact where more axillary lymph node metastases were seen in the OM group compared with the NOM group. In addition, through various analyses, CA153, ApoA1, and Hb were found to be the risk factors for OM in IDC patients.

### 4.1 CA153

CA153, as a breast cancer‐related antigen, is derived from the mammary epithelial surface. Elevated CA153 could be seen in 30%‐50% of breast cancer patients. However, its positive rate was only 20%‐30% in patients with early breast cancer. In spite of this, it is valuable in detecting distant metastases in breast cancer patients, especially when the disease cannot be evaluated by radiological procedures.^13^ The specificity and sensitivity of CA153 in predicting bone metastasis were 76.62% and 86.97%, respectively.^6^ In liver metastasis, they were 52.0% and 80.8%, respectively.^5^ In the present retrospective study, CA153 showed a high sensitivity (94.74%) in diagnosing OM in IDC patients. In addition, the cutoff value of CA153 was 43.3 u/mL, indicating that patients with IDC with a level of CA153 higher than 43.3 u/mL were more prone to develop OM.

### 4.2 ApoA1

ApoA1, as the main structural protein of HDL, participates in the reverse transport of cholesterol. Previous studies have shown that ApoA1 levels are associated with cardiovascular diseases.^14^ In addition, it has anti‐tumor functions. An experiment showed that the injection of ApoA1 into mice could effectively inhibit tumor growth and metastasis.^15^ Moreover, ApoA1 was found to have an effective immunomodulatory role in the tumor microenvironment, for it can change tumor‐associated macrophages from pre‐tumor M2 to anti‐tumor M1 phenotype.^15^

Studies have also shown decreased ApoA1 levels in patients with malignant tumors. For instance, ApoA1 levels were decreased in children with acute lymphoblastic leukemia and returned to normal after therapy.^16^ Investigations also suggested that people with low levels of ApoA1 are more likely to have lung cancer and colorectal carcinoma.^17^, ^18^ As it exerts anti‐tumor effects, ApoA1 has been used to diagnose and evaluate the prognosis of tumors. Farias‐Eisner et al.^19^ reported that ApoA1 combined with prealbumin and transferrin has a sensitivity of 71% in diagnosing endometrial cancer. Tuft et al.^20^ found that patients with ovarian serous adenocarcinoma have longer overall survival when higher levels of ApoA1 mRNA are detected in their chest and abdominal dropsy before chemotherapy. In the present retrospective study, IDC patients with ApoA1 lower than 1.11 g/L were prone to develop OM. It can be used to diagnose OM in patients with IDC, with a sensitivity of 84.21% and specificity of 94.88%.

### 4.3 Hemoglobin

Hb is a special protein in red blood cells, whose main function is to transport oxygen and carbon dioxide. Cancer patients are prone to have relatively low Hb levels due to tumor‐related hemorrhage, surgery, chemoradiotherapy, etc. With the decreasing of Hb, the oxygen partial pressure of tumor tissue also decreased significantly,^21^ which would cause hypoxia in tumor tissue. Hypoxia will increase the genetic instability of tumor cells and cause genomic changes,^22^ which will enhance tumor adaptability and give birth to some invasive tumor cells, leading to tumor progression and distant metastasis. Moreover, the high expression of hypoxia‐inducible factor‐1α (HIF‐1α), a crucial transcription factor, will cause angiogenesis of tumor tissue and accelerate tumor infiltration,^23^ which could easily lead to OM. In addition, hypoxia will also cause alterations of PI3 K/AKT/mTOR, MAPK, and NF‐ĸB pathways,^24^, ^25^, ^26^ thus promoting tumor metastasis. Hence, Hb level is closely related to the prognosis of cancer patients.

Currently, Hb level has been used as a prognostic factor in nasopharyngeal carcinoma, vulvar squamous cell carcinoma, advanced head and neck cancer, and early breast cancer.^24^, ^27^, ^28^, ^29^

In the present study, we found Hb an independent risk factor of OM in patients with IDC. IDC patients with Hb level lower than 112 g/L were more likely to develop OM. Therefore, Hb could also be used to predict OM in patients with IDC, with a sensitivity of 78.95% and specificity of 77.92%.

### 4.4 Combined diagnostic values

Moreover, to obtain a better predictive method, diagnostic values of the combined risk factors were also detected. Among the two combined risk factors, CA153 and ApoA1 had the best accuracy, with the sensitivity and specificity of 89.47% and 97.78%, respectively. The best diagnostic accuracy was obtained when using three combined risk factors, CA153+ApoA1+Hb, with the sensitivity and specificity of 89.47% and 97.78, respectively.

### 4.5 Axillary lymph node metastases

Lymph node metastases serve as a marker of the host response to malignant tumors, and the number of lymph node metastases indirectly reflects the invasiveness of tumors. In the study,^30^ it was found that compared with patients without lymph node metastases, those with four or more lymph node metastases had a worse prognosis after recurrence. However, the prognosis of patients with only one to three lymph nodes involved was not significantly different from that of patients with negative lymph node metastases, so the number of lymph nodes involved is a key determinant of the prognosis after recurrence. Bone is the most common part for distant metastases of breast cancer, whose most common extra‐organ metastasis is bone metastasis. In the study of Chen et al,^6^ it was found that lymph node metastasis could be an independent risk factor of breast cancer bone metastasis, and the incidence of breast cancer bone metastasis was the highest among patients with four or more axillary lymph nodes involved. Although there is no literature reporting that axillary lymph nodes can be taken as a risk factor of ocular metastasis from breast cancer, we can speculate that the number of axillary lymph node metastases might be an important risk factor for it.

We get most information through our eyes. Ocular metastasis of breast cancer is not very common, but once the tumor metastasizes to eyes, the patient's vision will be greatly affected, which will further reduce his quality of life. Therefore, improving the cognition of clinical features and risk factors of ocular metastasis among breast cancer patients is of great significance for early diagnosis and treatment, which can help improve the long‐term prognosis of patients and prevent serious consequences of ocular metastasis. A serum tumor examination has advantages including reproducibility, noninvasiveness, and a low cost, which is more convenient and quick compared with traditional CT and MRI, and it causes less harm to patients. Therefore, we analyzed the clinical characteristics of patients with ocular metastases from breast cancer and identified risk factors of ocular metastases.

### 4.6 Limitations

Yet, there were also some limitations in this retrospective study. First, some patients might have other distant metastases where elevated CA153 could also be seen,^31^ which might influence the conclusion. Second, the sample size of the OM group was relatively small as it is rare to be seen in clinical practice and all participants were from the same institution, which might have some bias. Third, the statistical analysis could only demonstrate a connection between risk factors and OM in IDC patients but the specific mechanisms still need further experiments.

To sum up, we determined the risk factors for OM in IDC and analyzed their diagnostic values. In clinical practice, when CA153, ApoA1 or Hb levels of IDC patients alter beyond their cutoff values, CT, MRI, and ocular ultrasound are advised to be performed to detect whether OM has occurred in IDC patients so that timely treatment could be made at an early stage which may improve patients' survival time and quality of life.

## 5 PATIENT CONSENT FOR PUBLICATION

Not applicable.

## CONFLICT OF INTEREST

The authors declare that they have no competing interests.

## AUTHORS' CONTRIBUTIONS

Shao Y designed the study. Ge QM, Liang RB, and Zhang YQ collected the clinical data. Fang JW and Liu JX performed statistical analyses. Fang JW, Min YL, and Li B prepared the manuscript. Lin Q and Shi WQ made the figures and tables. All authors read and approved the final manuscript.

## ETHICAL STATEMENT

This study was approved by the Medical Research Ethics Committee of the First Affiliated Hospital of Nanchang University and followed the principles of the Declaration of Helsinki. Informed consents from participants were obtained.

## References

[1] Li CI , Anderson BO , Daling JR , Moe RE . Trends in incidence rates of invasive lobular and ductal breast carcinoma. JAMA. 2003;289:1421‐1424. 12636465 10.1001/jama.289.11.1421

[2] Bray F , Ferlay J , Soerjomataram I , Siegel RL , Torre LA , Jemal A . Global cancer statistics 2018: GLOBOCAN estimates of incidence and mortality worldwide for 36 cancers in 185 countries. CA Cancer J Clin. 2018;68:394‐424. 30207593 10.3322/caac.21492

[3] Cohen VM . Ocular metastases. Eye. 2013;27:137‐141. 23222564 10.1038/eye.2012.252 PMC3574252

[4] Slimane K , Andre F , Delaloge S , et al. Risk factors for brain relapse in patients with metastatic breast cancer. Ann Oncol. 2004;15:1640‐1644. 15520065 10.1093/annonc/mdh432

[5] Cao R , Wang LP . Serological diagnosis of liver metastasis in patients with breast cancer. Cancer Biol Med. 2012;9:57‐62. 23691457 10.3969/j.issn.2095-3941.2012.01.011 PMC3643646

[6] Chen WZ , Shen JF , Zhou Y , Chen XY , Liu JM , Liu ZL . Clinical characteristics and risk factors for developing bone metastases in patients with breast cancer. Sci Rep. 2017;7:11325. 28900285 10.1038/s41598-017-11700-4 PMC5595860

[7] Hu J , Li G , Zhang P , Zhuang X , Hu G . A CD44v(+) subpopulation of breast cancer stem‐like cells with enhanced lung metastasis capacity. Cell Death Dis. 2017;8:e2679. 28300837 10.1038/cddis.2017.72 PMC5386565

[8] Sachs N , de Ligt J , Kopper O , et al. A Living Biobank of Breast Cancer Organoids Captures Disease Heterogeneity. Cell. 2018;172:373‐386. 29224780 10.1016/j.cell.2017.11.010

[9] Chen XY , Lan M , Zhou Y , et al. Risk factors for bone metastasis from renal cell cancer. J Bone Oncol. 2017;9:29‐33. 29159028 10.1016/j.jbo.2017.10.004 PMC5684431

[10] Huang P , Lan M , Peng AF , et al. Serum calcium, alkaline phosphotase and hemoglobin as risk factors for bone metastases in bladder cancer. PLoS One. 2017;12:e0183835. 28902911 10.1371/journal.pone.0183835 PMC5597169

[11] Zhou Y , Yu QF , Peng AF , Tong WL , Liu JM , Liu ZL . The risk factors of bone metastases in patients with lung cancer. Sci Rep. 2017;7:8970. 28827719 10.1038/s41598-017-09650-y PMC5567132

[12] Zhu PW , Gong YX , Min YL , et al. The predictive value of high‐density lipoprotein for ocular metastases in colorectal cancer patients. Cancer Manag Res. 2019;11:3511‐3519. 31118776 10.2147/CMAR.S194637 PMC6503335

[13] Duffy MJ , Evoy D , McDermott EW . CA 15–3: uses and limitation as a biomarker for breast cancer. Clin Chim Acta. 2010; 411:1869‐1874. 20816948 10.1016/j.cca.2010.08.039

[14] Thompson A , Danesh J . Associations between apolipoprotein B, apolipoprotein AI, the apolipoprotein B/AI ratio and coronary heart disease: a literature‐based meta‐analysis of prospective studies. J Intern Med. 2006;259:481‐492. 16629854 10.1111/j.1365-2796.2006.01644.x

[15] Zamanian‐Daryoush M , Lindner D , Tallant TC , et al. The cardioprotective protein apolipoprotein A1 promotes potent anti‐tumorigenic effects. J Biol Chem. 2013;288:21237‐21252. 23720750 10.1074/jbc.M113.468967 PMC3774392

[16] Halton JM , Nazir DJ , McQueen MJ , Barr RD . Blood lipid profiles in children with acute lymphoblastic leukemia. Cancer. 1998; 83:379‐384. 9669823

[17] van Duijnhoven FJ , Bueno‐De‐Mesquita HB , Calligaro M , et al. Blood lipid and lipoprotein concentrations and colorectal cancer risk in the European Prospective Investigation into Cancer and Nutrition. Gut. 2011;60:1094‐1102. 21383385 10.1136/gut.2010.225011

[18] Borgquist S , Butt T , Almgren P , et al. Apolipoproteins, lipids and risk of cancer. Int J Cancer. 2016;138:2648‐2656. 26804063 10.1002/ijc.30013

[19] Farias‐Eisner G , Su F , Robbins T , Kotlerman J , Reddy S , Farias‐Eisner R . Validation of serum biomarkers for detection of early‐ and late‐stage endometrial cancer. Am J Obstet Gynecol. 2010;202(73):e1‐e5. 10.1016/j.ajog.2009.07.049 19766980

[20] Tuft Stavnes H , Nymoen DA , Hetland Falkenthal TE , Kaern J , Trope CG , Davidson B . APOA1 mRNA expression in ovarian serous carcinoma effusions is a marker of longer survival. Am J Clin Pathol. 2014;142:51‐57. 24926085 10.1309/AJCPD8NBSHXRXQL7

[21] Kelleher DK , Mattheinsen U , Thews O , Vaupel P . Blood flow, oxygenation, and bioenergetic status of tumors after erythropoietin treatment in normal and anemic rats. Cancer Res. 1996;56:4728‐4734. 8840991

[22] Bristow RG , Hypoxia HRP . Hypoxia and metabolism. DNA repair and genetic instability. Nat Rev Cancer. 2008;8:180‐192. 18273037 10.1038/nrc2344

[23] Nagaraju GP , Bramhachari PV , Raghu G , El‐Rayes BF . Hypoxia inducible factor‐1alpha: Its role in colorectal carcinogenesis and metastasis. Cancer Lett. 2015;366:11‐18. 26116902 10.1016/j.canlet.2015.06.005

[24] Henke M , Sindlinger F , Ikenberg H , Gerds T , Schumacher M . Blood hemoglobin level and treatment outcome of early breast cancer. Strahlenther Onkol. 2004;180:45‐51. 14704844 10.1007/s00066-004-1123-7

[25] Royds JA , Dower SK , Qwarnstrom EE , Lewis CE . Response of tumour cells to hypoxia: role of p53 and NFkB. Mol Pathol. 1998;51:55‐61. 9713587 10.1136/mp.51.2.55 PMC395611

[26] Richard DE , Berra E , Gothie E , Roux D , Pouyssegur J . p42/p44 mitogen‐activated protein kinases phosphorylate hypoxia‐inducible factor 1alpha (HIF‐1alpha) and enhance the transcriptional activity of HIF‐1. J Biol Chem. 1999;274:32631‐32637. 10551817 10.1074/jbc.274.46.32631

[27] Guo SS , Tang LQ , Chen QY , et al. Is hemoglobin level in patients with nasopharyngeal carcinoma still a significant prognostic factor in the era of intensity‐modulated radiotherapy technology? PLoS One. 2015;10:e0136033. 26313452 10.1371/journal.pone.0136033 PMC4552389

[28] van de Nieuwenhof HP , de Hullu JA , Kaanders JH , Bulten J , Massuger LF , van Kempen LC . Hemoglobin level predicts outcome for vulvar cancer patients independent of GLUT‐1 and CA‐IX expression in tumor tissue. Virchows Arch. 2010;457:693‐703. 20890613 10.1007/s00428-010-0981-x PMC2995319

[29] Rudat V , Dietz A , Schramm O , et al. Prognostic impact of total tumor volume and hemoglobin concentration on the outcome of patients with advanced head and neck cancer after concomitant boost radiochemotherapy. Radiother Oncol. 1999;53:119‐125. 10665788 10.1016/s0167-8140(99)00119-x

[30] Jatoi I , Hilsenbeck SG , Clark GM , Osborne CK . Significance of axillary lymph node metastasis in primary breast cancer. J Clin Oncol. 1999;17(8):2334. 10561295 10.1200/JCO.1999.17.8.2334

[31] Arslan N , Serdar M , Deveci S , et al. Use of CA15‐3, CEA and prolactin for the primary diagnosis of breast cancer and correlation with the prognostic factors at the time of initial diagnosis. Ann Nucl Med. 2000;14:395‐399. 11108173 10.1007/BF02988705

